# Recombinant SARS-CoV-2 Nucleocapsid Protein: Expression, Purification, and Its Biochemical Characterization and Utility in Serological Assay Development to Assess Immunological Responses to SARS-CoV-2 Infection

**DOI:** 10.3390/pathogens10081039

**Published:** 2021-08-16

**Authors:** Da Di, Mythili Dileepan, Shamim Ahmed, Yuying Liang, Hinh Ly

**Affiliations:** Department of Veterinary and Biomedical Sciences, College of Veterinary Medicine, University of Minnesota, St Paul, MN 55108, USA; ddi@umn.edu (D.D.); dilee002@umn.edu (M.D.); ahme0242@umn.edu (S.A.)

**Keywords:** COVID-19, SARS-CoV-2, nucleocapsid, RNA-binding protein, ELISA, diagnostics

## Abstract

The SARS-CoV-2 nucleocapsid protein (N) binds a single-stranded viral RNA genome to form a helical ribonucleoprotein complex that is packaged into virion particles. N is relatively conserved among coronaviruses and consists of the N-terminal domain (NTD) and C-terminal domain (CTD), which are flanked by three disorganized regions. N is highly immunogenic and has been widely used to develop a serological assay as a diagnostic tool for COVID-19 infection, although there is a concern that the natural propensity of N to associate with RNA might compromise the assay’s specificity. We expressed and purified from bacterial cells two recombinant forms of SARS-CoV-2 N, one from the soluble fraction of bacterial cell lysates that is strongly associated with bacterial RNAs and the other that is completely devoid of RNAs. We showed that both forms of N can be used to develop enzyme-linked immunosorbent assays (ELISAs) for the specific detection of human and mouse anti-N monoclonal antibodies (mAb) as well as feline SARS-CoV-2 seropositive serum samples, but that the RNA-free form of N exhibits a slightly higher level of sensitivity than the RNA-bound form to react to anti-N mouse mAb. Using the electrophoretic mobility shift assay (EMSA), we also showed that N preferentially binds ssRNA in a sequence-independent manner and that both NTD and CTD of N contribute to RNA-binding activity. Collectively, our study describes methods to express, purify, and biochemically characterize the SARS-CoV-2 N protein and to use it for the development of serological assays to detect SARS-CoV-2 infection.

## 1. Introduction

SARS-CoV-2, a novel severe acute respiratory syndrome (SARS)-related coronavirus (lineage 2B, genus *Betacoronavirus*), is the causative agent of the COVID-19 pandemic that is closely related to SARS-CoV and a bat CoV [1,2]. Monoclonal antibodies (mAbs) can be used to treat mild to moderate COVID-19 cases but not severe disease [3]. FDA has approved several vaccines for emergency use authorization (EUA) [4], which include Pfizer/BioNTech’s and Moderna’s mRNA-based vaccines and Johnson & Johnson’s adenoviral-vector-based vaccine.

SARS-CoV-2 is an enveloped RNA virus with a large (~30 kb) positive-sense RNA genome that encodes 16 nonstructural proteins (NSPs), 4 structural proteins, and multiple accessory proteins called ORF3–ORF10 [5]. The structural proteins include spike (S), envelope (E), membrane (M), and nucleocapsid (N). The N protein encapsidates viral genomic RNA and forms a helical ribonucleoprotein that is packaged into virion particles [6,7]. In addition to viral RNA packaging and viral particle formation [8], N has been shown to participate in subgenomic mRNA transcription by recruiting the RNA helicase DDX1 to facilitate RNA template readthrough [9].

N is one of the most abundantly expressed and relatively conserved proteins among coronaviruses (CoVs) [10] and is one of the major targets of antibody development upon CoV infections [11], and therefore, it had been used in the development of serological diagnostic tools for COVID-19 infection [12,13]. Due to its high level of immunogenicity and potential to induce cross-reactive immune responses, the N protein is also considered another major target for vaccine development (besides the viral spike protein), although experimental evidence for its protective immunity is still lacking [10].

The N protein of SARS-CoV-2 has over 90% sequence similarity with SARS and ~60% similarity with MERS, another human CoV pathogen within the betacoronavirus genus. All CoV N proteins share a similar domain architecture [14], consisting of the highly conserved N-terminal domain (NTD) and C-terminal domain (CTD), which are flanked by three disordered regions [15]. NTD, from aa 40 to 180 of SARS-CoV-2 N, is the RNA-binding domain, while CTD, from aa 247 to 364 of SARS-CoV-2 N, is the protein dimerization domain that binds RNA [16,17,18,19,20]. Three disordered regions of SARS-CoV-2 N are located around aa 1–40, aa 180–247, and aa 365–418, whose functions have not been clearly defined. These regions consist of many basic amino acid residues that may facilitate RNA binding [14]. Cubuk and colleagues recently performed single-molecule spectroscopy coupled with all-atom simulations to reveal minimal interaction between NTD and CTD of SARS-CoV-2 N that affords the flexibility and multivalent RNA-binding activity of the full-length N protein and allows it to undergo a liquid–liquid phase separation when mixed with RNA [21].

While the structures of NTD and CTD for SARS-CoV-2 have been determined [16,17,18], they are not shown in complex with nucleic acids. The structures of the full-length N proteins of SARS-CoV and SARS-CoV-2 are not yet available, partly because of the technical difficulty of purifying these proteins. Some efforts have recently been made to express and purify the full-length coronaviral N proteins. For example, Timani and colleagues successfully purified the N protein of SARS-CoV for use in developing a serological assay [22]. A similar effort was made to purify the full-length and N-terminally truncated versions of the SARS-CoV-2 N protein for use in serological assay development [23]. The authors of this work suggest that the N-terminally truncated protein is a better serological marker than the full-length protein for use in assessing the immunogenicity of inactivated SARS-CoV-2. It is worth noting that neither of the studies determine whether the purified SARS-CoV and SARS-CoV-2 N proteins retain the ability to bind to nucleic acids. Zeng and colleagues successfully purified the SARS-CoV-2 N protein from bacteria and showed that it existed largely in dimeric form and had a natural tendency to bind to nucleic acid nonspecifically [24]. The authors raised a real concern that this form of the N protein (i.e., N(+RNA)) might have a negative impact when used as a diagnostic marker. It is therefore important to carefully assess the validity of serological assays that involve the use of recombinant SARS-CoV-2 N proteins with or without nucleic acids.

In this study, we expressed and purified from bacteria two different forms of recombinant SARS-CoV-2 N, a soluble form that is strongly associated with bacterial RNAs and another that is completely free of RNAs. We used these two forms of N for the development of serological assays to compare their specificity and sensitivity against a known human monoclonal antibody (mAb) of SARS-CoV-2 and used the more sensitive assay to survey cat sera collected from the Veterinary Medical Center at the University of Minnesota for evidence of potential exposure to SARS-CoV-2. Using a biochemical assay, we also found that the recombinant N protein preferentially binds to ssRNA, which is consistent with its expected role in encapsidating a viral ssRNA genome during the authentic viral life cycle.

## 2. Results

### 2.1. Recombinant SARS-CoV-2 N Protein from the Soluble Fraction of Bacterial Cell Lysates Contains Bacterial RNAs

**Figure 1:**
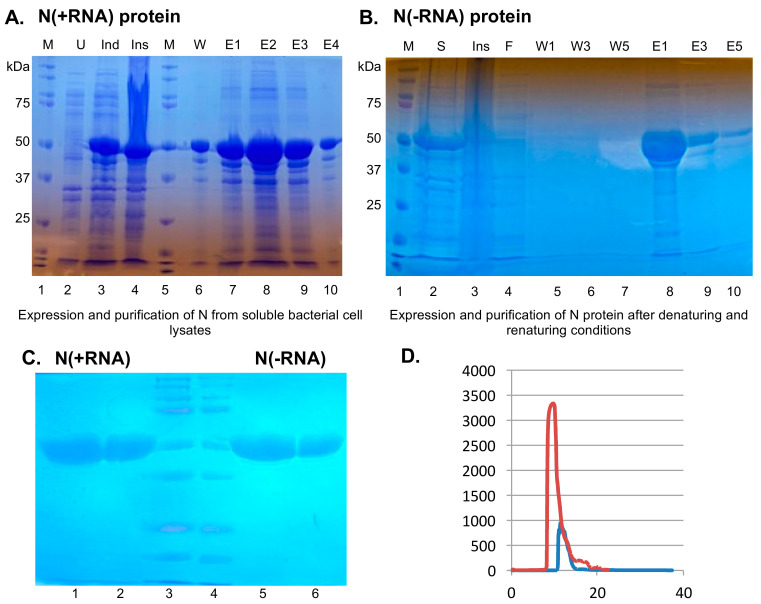
Expression and purification of the recombinant SARS-CoV-2 N protein with or without associated bacterial RNAs. Bacterial cell lysates, fractions, and wash and elution collections were loaded onto and separated by 12% SDS-PAGE, followed by Coomassie blue staining. (**A**) Expression and purification of the SARS-CoV-2 N protein from the soluble bacterial cell lysates. M, protein markers. U, uninduced cell lysates. Ind, IPTG-induced cell lysates. Ins, insoluble fraction. W, solution after washing the HisTrap column. E1 to E4, elution fractions. (**B**) Purification of the SARS-CoV-2 N protein using the denaturation and renaturation methods. M, protein markers. S, soluble fraction after protein denaturation by urea treatment. Ins, insoluble protein fraction after urea denaturation. F, flow-through fraction after applying proteins through the HisTrap column. W1, W3, and W5 fraction collections after washing the HisTrap column. E1, E3, and E5 fraction collections after elution of the samples from the HisTrap column. (C) Detection of the purified N(+RNA) and N(−RNA) proteins by gel electrophoresis after Coomassie blue staining. N(+RNA) at 15 mg (lane 1) and 5 mg (lane 2), N(−RNA) at 15 mg (lane 5) and 5 mg (lane 6). M, same molecular markers in lanes 3 and 4 of panel (**C**) as shown in lanes 1 and 5 of panel (**A**) and in lane 1 of panel (**B**). (**D**) Gel filtration of N(+RNA) shown in red color and of N(−RNA) shown in blue color. *X*-axis represents column retention volumes, and *Y* axis shows UV absorbance values at 280 nm wavelength.

We expressed and purified a recombinant SARS-CoV-2 N protein from the soluble fractions of bacterial cell lysates by culturing the N-protein plasmid containing bacterial cells at a low temperature (16 °C) and with a relatively low concentration of IPTG (0.1 mM). We analyzed the expression level of the recombinant N protein at each step of the expression and purification process via 12% SDS-PAGE (Figure 1A). When compared with the uninduced condition that produced no SARS-CoV-2 N (Figure 1A, lane 2), the recombinant N protein expressed at a relatively high level upon IPTG induction (Figure 1A, lane 3) and remained largely in the insoluble fraction (Figure 1A, lane 4) of the bacterial cell lysates. The recombinant N protein was also present in the soluble fraction, which was purified by the HisTrap affinity column (Figure 1A, lanes 7 to 9). About 2 mg of the recombinant SARS-CoV-2 N protein could be purified from 1 L of the bacterial cell culture. The OD260/280 ratio of the protein sample was 1.84, which suggests that the recombinant SARS-CoV-2 N protein was strongly associated with nucleic acids. RNase A and RNase III treatments of the soluble fraction of the cell lysates prior to the protein purification step did not reduce the presence of nucleic acids in the final protein product, suggesting that nucleic acids are likely integral components of the recombinant SARS-CoV-2 N protein, which is consistent with its function as the nucleocapsid protein that encapsidates viral genomic ssRNAs [6,7]. For convenience, this form of the SARS-CoV-2 N protein that is intimately associated with bacterial RNAs shall henceforth be referred to as N(+RNA).

### 2.2. Purification of Recombinant SARS-CoV-2 N Protein Devoid of Bacterial RNAs

In order to biochemically characterize the RNA-binding activity of the SARS-CoV-2 N protein, we must develop a method to purify it from bacterial RNAs. Previous studies with the SARS-CoV N protein showed that this could be accomplished by chemically inducing protein unfolding by treatment with urea or guanidine hydrochloride, after which the recombinant protein could then be refolded to its native state and was free of any RNAs [25,26]. Since most of the recombinant SARS-CoV-2 N protein was present in the insoluble fraction (i.e., inclusion body) of the bacterial cell lysates, we decided to use a denaturing buffer that contains a relatively high concentration (6 M) of urea in order to solubilize the inclusion body and to denature the recombinant SARS-CoV-2 N protein (Figure 1B, lane 2). The unfolded recombinant protein was then applied to the HisTrap affinity column and washed to remove any protein contaminants (Figure 1B, lanes 4–7). The recombinant SARS-CoV-2 N protein was then refolded in a renaturing buffer and eluted from the column (Figure 1B, lanes 8–10). For convenience, we henceforth refer to it as N(−RNA). The OD260/280 ratio indicated that this recombinant SARS-CoV-2 N protein was free of nucleic acids (data not shown). To validate the quality of the purified proteins, we analyzed the N(+RNA) and N(−RNA) proteins side by side in different concentrations on SDS-PAGE. Coomassie blue staining detected a major band of 50 kDa for both recombinant proteins in a dose-dependent manner (Figure 1C). Additional minor bands of lower molecular weight were consistently detected in both forms of the proteins, but at minute quantities, suggesting that both forms of the N proteins can be purified at a relatively high quality and quantity. About 150 mg of the RNA-free form of the recombinant SARS-CoV-2 N protein (N(−RNA)) could be purified from 1 L of the bacterial cell culture. To validate the purity of N(+RNA) and N(−RNA), we collected gel filtration data of both forms of the protein (Figure 1D) and showed that they both existed mainly as major products that eluded between column retention volumes 5 and 15, but that some smaller amounts of the proteins could also be seen at higher column retention volumes (mainly in the N(+RNA) sample), suggesting the presence of some relatively minor amounts of N protein impurity and/or multimerization. It is noteworthy that N(+RNA) shows a much higher UV absorbance value than the N(−RNA) sample, indicating the presence of nucleic acids in the N(+RNA) sample, as expected.

### 2.3. Both N(+RNA) and N(−RNA) Proteins Can Be Specifically Detected by an Anti-N Antibody

**Figure 2:**
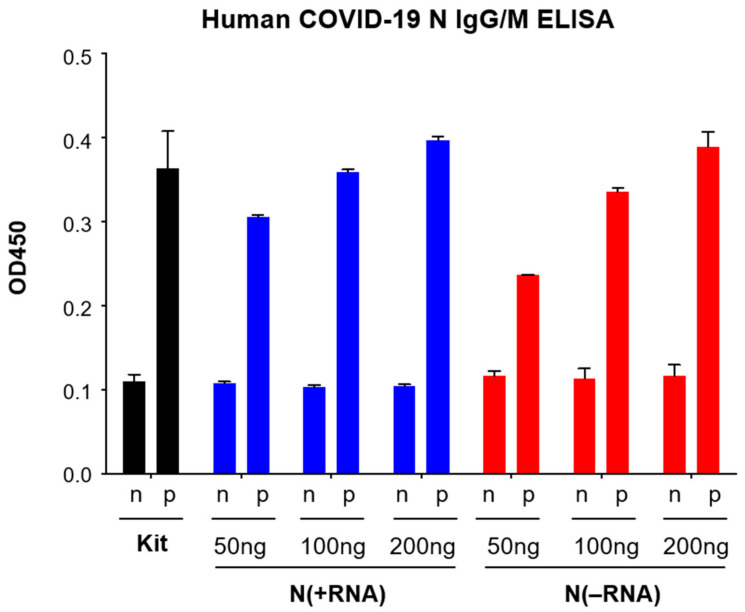
Evaluation of the recombinant N(−RNA) and N(+RNA) proteins to detect human anti-SARS-CoV-2 N mAb by a commercial human COVID-19 N IgG/IgM ELISA kit. An ELISA plate was coated with the N(−RNA) or N(+RNA) protein at indicated concentrations per well and incubated with the positive (p) or negative (n) controls supplied by the commercial COVID-19 N IgG/IgM ELISA kit. Results shown are the average of triplicate sets of data at different SARS-CoV-2 N protein concentrations. At least two independent experiments were conducted for each set of experiments. Results obtained from the commercial kit are shown in black bars, those from N(+RNA)-coated wells are shown in blue bars, and those from N(−RNA)-coated wells in red bars. p, positive control; n, negative control.

We next attempted to use N(+RNA) and N(−RNA) proteins to develop an ELISA using reagents in a commercial COVID-19 N-based human IgG/IgM ELISA kit (MyBioSource, cat. no. MBS3809905). Toward this end, we coated ELISA plates with the N(+RNA) or N(−RNA) protein at different concentrations (50, 100, and 200 ng per well) and used the positive (human anti-N antibody) and negative controls supplied in the kit in the recommended procedure. Both N(−RNA) and N(+RNA) at different protein concentrations produced consistently low background OD_450_ values with the negative control (n) and significantly high OD_450_ values with the positive control (p) in a dose-dependent manner (Figure 2). One hundred nanograms (100 ng) of the recombinant SARS-CoV-2 N protein, with or without the associated RNAs, produced similar OD_450_ values as those present in the precoated plate of the commercial kit. This demonstrates that both forms of the recombinant SARS-CoV-2 N proteins can be specifically recognized by an anti-COVID-19 N antibody.

### 2.4. N(−RNA) Shows Higher Sensitivity Level Than N(+RNA) to Detect Anti-N Antibody by ELISA

**Figure 3:**
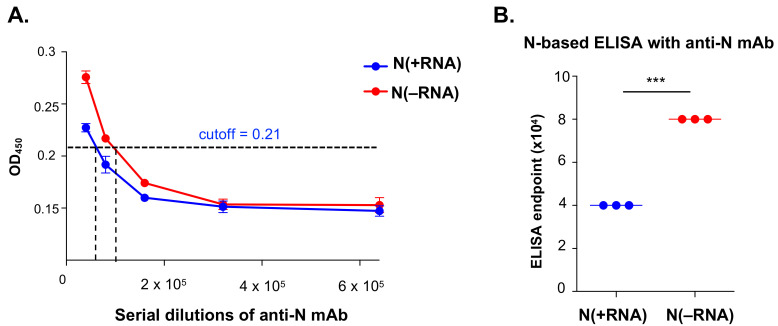
Evaluation of N(+RNA) and N(−RNA) proteins in detecting anti-N mAb by ELISA. Serial dilutions of mouse anti-SARS-CoV N mAb were incubated in an ELISA plate precoated with 100 mg of either N(−RNA) or N(+RNA) and analyzed by standard ELISA procedure as described in Materials and Methods. (**A**) The average OD_450_ value of triplicates is plotted against the mAb dilutions for N(−RNA) or N(+RNA). The cutoff value is shown as a dashed line. (**B**) The N-specific IgG endpoint titers, based on N(−RNA) and N(+RNA), were calculated as the highest dilution to give an OD_450_ value exceeding the cutoff OD_450_ value. *** *p* ≤ 0.001.

To compare the sensitivity levels of the N-based ELISAs that are based on N(−RNA) and N(+RNA), we applied serial dilutions of a mouse anti-SARS-CoV N mAb (as a positive control) into N(−RNA)- and N(+RNA)-coated wells, each in triplicate. Shown in Figure 3A are the OD_450_ values plotted against the mAb dilutions, of which the same dilutions of mAb produced higher OD_450_ values in N(−RNA)-coated wells than in N(+RNA)-coated ones. After calculating the cutoff value at 0.21, the endpoints of the anti-N mAb were 4 × 10^4^ when N(+RNA) was used and 8 × 10^4^ when N(−RNA) was used (Figure 3B), demonstrating that N(−RNA) has a slightly higher level of sensitivity than N(+RNA) in detecting anti-N antibodies.

**Figure 4:**
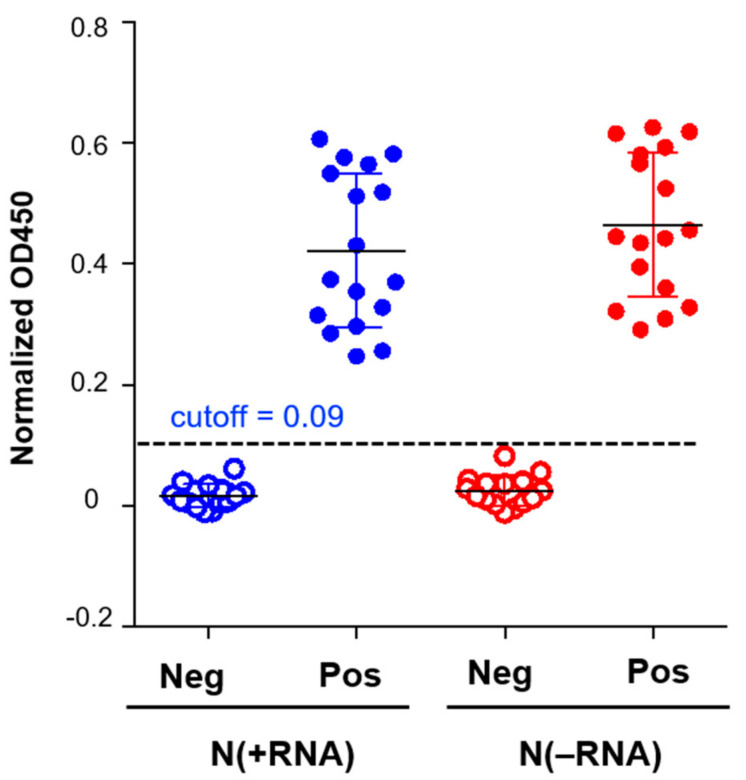
Evaluation of N(+RNA) and N(−RNA) proteins in the N-based ELISA using feline clinical serum samples. Seventeen seropositive (pos) and 14 seronegative (neg) cat serum samples were analyzed by N-based ELISA with either N(+RNA) and N(−RNA). The cutoff OD_450_ value is shown as a dashed line.

To compare N(−RNA) and N(+RNA) in the application of an N-based IgG ELISA to detect anti-SARS-CoV-2 N antibody in clinical serum samples, we used 31 cat serum samples, including 17 seropositive and 14 seronegative ones, that were previously confirmed by both the SARS-CoV-2 receptor-binding domain (RBD) and N-based IgG ELISAs as well as in an established COVID-19 neutralization assay [27]. Each cat serum sample was added in an equal amount into the wells of a 96-well plate and tested in an ELISA plate under three conditions, uncoated, N(+RNA) coated, and N(−RNA) coated. The adjusted OD_450_ value was calculated by subtracting the value in the uncoated well from that in the respective N-coated well. Both N(+RNA)- and N(−RNA)-based IgG ELISAs produced consistently low OD_450_ values for all negative samples and significantly higher OD_450_ values than the cutoff value at 0.09 for all positive samples (Figure 4). The results demonstrate that both N(+RNA) and N(−RNA) proteins can be used to detect anti-N antibodies in clinical serum samples with similar specificity and sensitivity.

### 2.5. The SARS-CoV-2 N Protein Preferentially Binds ssRNA In Vitro

**Figure 5:**
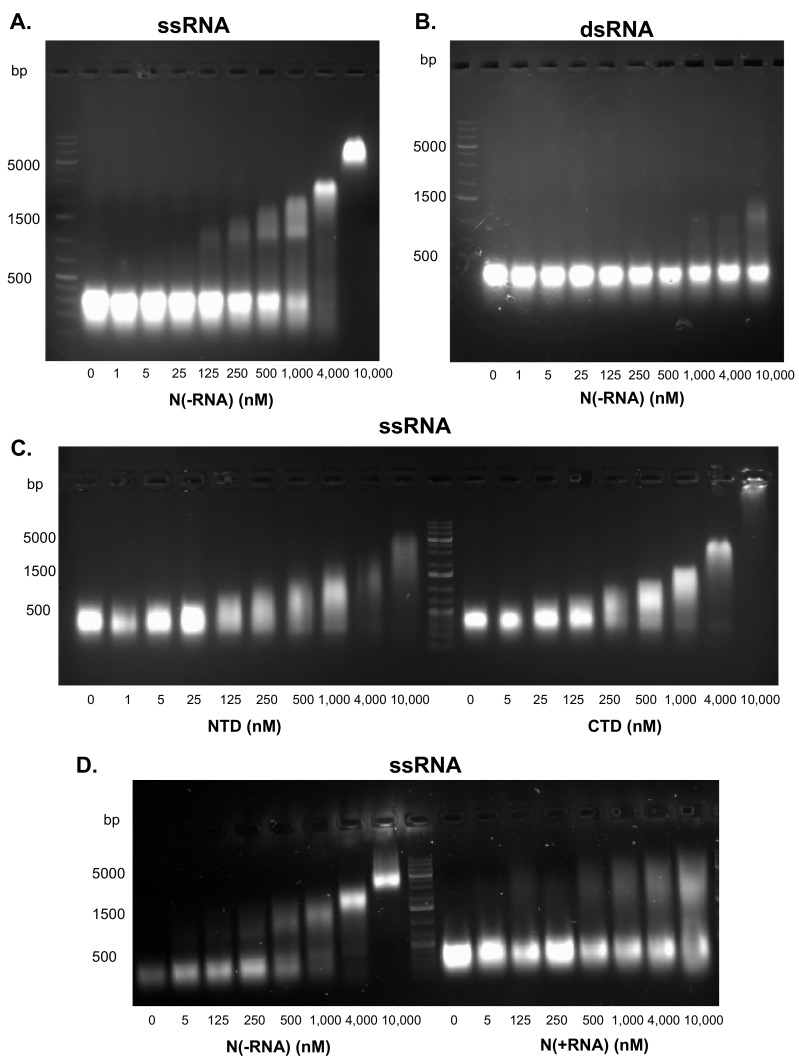
The recombinant SARS-CoV-2 N protein preferentially binds to ssRNA in vitro. The ssRNA (**A**) and dsRNA (**B**) templates were incubated with increasing concentrations of the recombinant N(−RNA) protein and separated in 1% agarose gel with ethidium bromide. The ssRNA template was incubated with either NTD or CTD of the SARS-CoV-2 N protein (**C**), with either N(−RNA) or N(+RNA) protein (**D**), at increasing concentrations and separated in 1% agarose gel with ethidium bromide. At least two independent experiments were conducted per assay. Molecular markers were loaded into the well either on the left or near the center of gels.

A major function of the CoV N protein is to encapsidate a viral ssRNA genome. To determine whether the recombinant N(−RNA) protein is functional in its RNA-binding activity, we conducted EMSA. Single-stranded or double-stranded RNA templates were incubated with increasing concentrations of N(−RNA) and separated on 1% agarose gel. N(−RNA) was shown to bind ssRNA in a concentration-dependent manner (Figure 5A). The shifted RNA-protein band was first detected at 125 nM of the N(−RNA) protein and increased in its intensity and size along with increasing N(−RNA) protein concentrations, suggesting multimerization of the N(−RNA) with the RNA substrate. At the highest concentration of the N(−RNA) protein (10 uM), however, all ssRNA molecules shifted to form a discrete high-molecular-weight band, suggesting that all ssRNAs were bound by the same number of N(−RNA) proteins (Figure 5A). In contrast, the dsRNA templates did not show any detectable shifted products until the N(−RNA) proteins were at relatively high concentrations, but a significant portion of the dsRNA substrates did not associate with any N(−RNA) protein even at the highest concentration (10 uM) (Figure 5B), strongly suggesting that the recombinant N(−RNA) protein preferentially binds to ssRNA.

We also expressed and purified NTD and CTD of the N(−RNA) protein and characterized their RNA-binding activities using EMSA. Both NTD and CTD were able to shift ssRNA substrates in a concentration-dependent manner (Figure 5C), demonstrating that both protein domains have RNA-binding activity, which is consistent with previous findings with CoV N proteins [16,17,18,19,20]. To determine whether N(+RNA) retains the ability to bind the ssRNA template, we compared the N(−RNA) and N(+RNA) proteins in EMSA (Figure 5D). In contrast to the efficient RNA-binding activity of the N(−RNA) protein forming discrete high-molecular-weight bands on the gel, the N(+RNA) protein was very inefficient at forming any discrete high-molecular-weight products, as it failed to bind to a majority of the ssRNA templates (Figure 5D), demonstrating that the N(+RNA) protein cannot associate with more ssRNA templates than its naturally bound form.

## 3. Discussion

The CoV N protein has widely been used for serological diagnosis of viral infection due to its high level of immunogenicity [28] and can also be a good antiviral target due to the essential roles it plays in the viral life cycle and its sequence conservation among CoVs [29,30]. We successfully expressed and purified the recombinant SARS-CoV-2 N protein from bacterial cells in two different forms: a soluble N(+RNA) form that can strongly associate with bacterial RNAs and a refolded N(−RNA) protein that is free of RNAs. We used these two versions of the recombinant SARS-CoV-2 N proteins to conduct biochemical studies and to develop serological assays. Both forms of the recombinant SARS-CoV-2 N protein can be used to specifically detect anti-N antibodies (i.e., mAb and antiviral sera) with an N(−RNA) form, showing a slightly higher level of sensitivity than N(+RNA) (Figure 3). Given the high yield of the recombinant N(−RNA) produced in bacteria and its high levels of specificity and sensitivity in detecting anti-N antibodies, this N(−RNA) protein is an ideal product for use in developing an N-based serological assay to screen for SARS-CoV-2 infections. Given the increasingly high vaccination rates for COVID-19 in the general populations, the N-based COVID-19 serological assays provide a valuable tool to distinguish natural SARS-CoV-2 infection from vaccination, because all current vaccine formulations use the viral spike (S) protein but not N as an antigen. Using the N(−RNA), we set up a new N-based IgG ELISA to screen a relatively small number of the already confirmed household cat sera as part of a larger study to survey pet cats and dogs admitted to the Veterinary Medical Center at the University of Minnesota that demonstrated a higher seroprevalence of SARS-CoV-2 infection of pet cats than of pet dogs in the early phase of the COVID-19 pandemic in the state of Minnesota [27].

Our biochemical assays suggest that the recombinant SARS-CoV-2 N protein that is expressed and purified in bacteria binds to RNA in a sequence-independent manner and is tightly associated with bacterial RNAs and is strongly resistant to RNase treatments. This is consistent with previous findings that CoV N encapsidates viral genomic ssRNA in a sequence-independent manner [31].

Previous studies also showed that CoV N has nonspecific binding activity toward nucleic acids [24,32] with CoV N binding to ssRNA being more specific than to DNA. For example, Tang and colleagues [32] showed that the ssRNA-N complexes resolved as a sharp and strong band in the EMSA gel, suggesting that the complexes are uniform in size and are resistant to RNase treatments, while the DNA-N complexes produced a smeary band on the EMSA gel, suggesting that these interactions are less specific [32]. In our current study, we showed that ssRNA-N interactions appeared to be more specific and stronger than those of the dsRNA-N complexes on the EMSA gels (Figure 5), suggesting that N binds to ssRNA more specifically than to dsRNA. While we have not determined the kinetics of RNA-N interactions, a recent prepublication (available in the bioRxiv archive) measured the binding affinity of SARS-CoV-2 N to ssRNA and stem-loop RNA (slRNA) and showed that the binding of SARS-CoV-2 N to slRNA is in an order of magnitude lower than that to ssRNA [33], and is therefore consistent with our current findings. It is also worth noting another prepublication [34] that describes a different method to dissociate the recombinant SUMO-tagged SARS-CoV-2 N protein from nucleic acids via solubilization coupled with heparin treatment and gel filtration chromatography.

While the exact mechanism of SARS-CoV-2 N’s RNA binding activity is still unclear, a previous study showed that SARS-CoV N binds to RNA cooperatively and requires multiple regions of the protein, such as NTD, CTD, and its disordered regions [14]. However, the exact mechanism by which CoV Ns encapsidate viral ssRNA genomes to form nucleocapsid is still poorly understood, partly due to the lack of an atomic structure for the full-length CoV N or any of its predicted domains in complex with nucleic acids. In addition to genome encapsidation, CoV N has recently been shown to participate in the regulation of viral RNA transcription by recognizing viral transcriptional regulatory sequences [35], suggesting that, under a certain condition, CoV N may harbor a sequence-specific RNA-binding activity.

In summary, we have reported findings on characterizing the biochemical activities of the recombinant SARS-CoV-2 N protein with or without the associated RNA by demonstrating its preferential ssRNA-binding activity in vitro and have developed a method to purify a recombinant SARS-CoV-2 N protein that is free of any RNA and used it to develop a serological assay to screen cat sera for evidence of SARS-CoV-2 infections. We believe that this new serological assay can serve as an important diagnostic tool for the detection of COVID-19 in humans, companion animals, and perhaps other animal species. Future structural and functional characterizations of the recombinant SARS-CoV-2 N are needed in order to facilitate the development of potential SARS-CoV-2 N-targeting antiviral agents.

## 4. Materials and Methods

### 4.1. Plasmids

The bacterial protein expression plasmid pRSF-Duet1 was used to clone the full-length SARS-CoV-2 N gene (GenBank accession number YP_009724397) that was synthesized by Twist Bioscience (San Francisco, CA, USA) with additional sequences to fuse the N protein with an N-terminal His tag and a C-terminal Strep tag. This plasmid is called pRSV-CoV2-N. The synthetic DNA sequences spanning the N-terminal domain (NTD) (44–180 aa) and C-terminal domain (CTD) (247 to 364 aa) of the N protein were individually cloned into the bacterial protein expression vectors to generate the pRSF-CoV2-N-NTD and pRSF-CoV2-N-CTD plasmids.

### 4.2. Expression and Purification of the Recombinant N Protein from the Soluble Fraction of Bacterial Cell Lysates

BL21(DE3) bacterial cells separately transformed with the different N protein expression plasmids were grown at 37 °C and prechilled at 4 °C. Isopropyl β-d-1-thiogalactopyranoside (IPTG) (Sigma-Aldrich, St. Louis, MO, USA) at a final concentration of 0.1 mM was added. The bacterial culture was incubated at 16 °C for 18 h. Bacterial cells were collected and resuspended in buffer (20 mM Na_2_HPO_4_, pH 7.4, 500 mM NaCl) and lysed by sonication. After centrifugation at 23,000× *g* for 60 min, the bacterial supernatants were filtered and applied onto a HisTrap HP column (GE healthcare, Chicago, IL, USA) using the AKTA pure chromatography system. At least 20 column volumes of the wash buffer (20 mM Na_2_HPO_4_, pH 7.4, 500 mM NaCl, 50 mM imidazole) were applied through the column before the proteins were eluted with 10 column volumes of the elution buffer (20 mM Na_2_HPO_4_, pH 7.4, 500 mM NaCl, 500 mM imidazole). The recombinant N protein’s fractions were pooled and concentrated using the Amicon Ultra-15 centrifugal filter unit (Millipore, Burlington, MA, USA; cat. # UFC900308). The proteins were analyzed for OD260/280 ratio by NanoDrop and quantified by the Bradford assay (Bio-Rad, Hercules, CA, USA).

### 4.3. Purification of the Recombinant N Protein without Associated Bacterial RNAs

BL21(DE3) bacterial cells separately transformed with the different N expression plasmids were grown at 37 °C overnight in the presence of 1 mM IPTG. The bacterial cells were collected and resuspended in the denaturing buffer (20 mM Na_2_HPO_4_, pH 7.4, 500 mM NaCl, and 6 M urea) and lysed by sonication. After centrifugation at 25,000× *g* for 60 min, the bacterial supernatants were collected, filtered, and applied onto the HisTrap HP column (GE healthcare) using the AKTA pure chromatography system. The same denaturing buffer was used to wash the column, which was followed by the application of a renaturing buffer (20 mM Na_2_HPO_4_, pH 7.4, 500 mM NaCl) and the HisTrap wash buffer (20 mM Na_2_HPO_4_, pH 7.4, 500 mM NaCl, 50 mM imidazole). Ten column volumes of the HisTrap elution buffer (20 mM Na_2_HPO_4_, pH 7.4, 500 mM NaCl, and 500 mM imidazole) were used to elute the recombinant N protein. Fractions that contained the N proteins were pooled and dialyzed against PBS buffer overnight. The proteins were analyzed for OD260/280 ratio by NanoDrop and quantified by the Bradford assay (Bio-Rad). The proteins were also analyzed by SDS-polyacrylamide gel electrophoresis (SDS-PAGE), followed by Coomassie blue staining and gel filtration (Superdex increase 10/300, 200 pg, GE Healthcare) analysis.

### 4.4. Electrophoretic Mobility Shift Assay (EMSA)

Single-stranded (ss) RNAs were generated from a PCR product that contains the 5′ T7 promoter and the first 218 nucleotides (nt) of the cDNA sequence of the Lassa virus L polymerase gene using the MEGAshortscript™ T7 Transcription Kit (Thermo Fisher Scientific, Waltham, MA, USA). The RNA products were treated with DNase I (New England Biolabs, Ipswich, MA, USA) before phenol/chloroform extraction and precipitation using ethanol and 0.3 M sodium acetate and dissolved in nuclease-free water (Thermo Fisher Scientific). To generate double-stranded (ds) RNAs, two ssRNAs were independently transcribed from the respective PCR products that contain the reverse complementary DNA sequences of the 218-nt Lassa virus L gene fragment under the T7 promoter, following the methods described above. The two reverse-complementary ssRNAs were then mixed at an equal concentration of 20 μM in annealing buffer (60 mM KCl, 10 mM HEPES pH 7.5, 0.2 mM MgCl_2_), heated at 95 °C for 2 min, and slowly cooled down to 25 °C at a rate of 1 °C per min.

To conduct EMSA, 1 mg of RNA templates (ssRNA or dsRNA) was incubated with increasing concentrations (from 0 to 10 μM) of the purified N proteins in a binding buffer (20 mM HEPES, 100 mM NaCl, 10% glycerol) at room temperature for 30 min. The samples were separated in 1% agarose gel with ethidium bromide using 1× Tris-acetic acid-EDTA (1× TAE) buffer and imaged by the myECL imager (Thermo Fisher Scientific).

### 4.5. Evaluation of the Recombinant SARS-CoV-2 N Protein Using Reagents of a Commercial Human IgG/IgM ELISA Kit

The recombinant SARS-CoV-2 N protein was evaluated using reagents in a commercial COVID-19 N-based human IgG/IgM ELISA kit (MyBioSource, San Diego, CA, USA; cat. no. MBS3809905). Specifically, a 96-well MaxiSorp ELISA plate (Thermo Fisher Scientific) was coated with the recombinant N with associated bacterial RNA, named N(+RNA), or without the associated RNA, named N(−RNA) at 50, 100, and 200 ng per well. The plate was incubated at 37 °C for 1 h, washed three times with phosphate-buffered saline containing 0.05% Tween 20 (i.e., PBST buffer), blocked with 5% nonfat milk in PBST at 37 °C for 1 h, and washed again three times with the same buffer. The positive and negative controls supplied in the kit were then added to the respective wells. ELISA was conducted following the manufacturer’s instructions.

### 4.6. Comparison of N(+RNA) and N(−RNA) in the N-Based ELISA with Serially Diluted Anti-N Monoclonal Antibody (mAb)

A 96-well MaxiSorp plate (Thermo Fisher Scientific, cat. no. 437111) was coated with 100 ng of the purified N(+RNA) or N(−RNA) protein overnight at 4 °C, blocked with 5% nonfat milk in PBST buffer at 37 °C for 1 h, and washed with PBST buffer as described above. PBS or anti-SARS-CoV N monoclonal antibody (a kind gift of L. Martinez-Sobrido, Texas Biomedical Research Institute) was added to N(+RNA)- and N(−RNA)-coated wells in serial twofold dilutions, each in triplicate, and incubated for 1 h at 37 °C. Following PBST washing, HRP-conjugated goat anti-mouse IgG polyclonal antibody (R&D Systems, cat. no. HAF007) was added at a 1:1000 dilution and incubated for 45 min at 37 °C. The substrate ABTS (Millipore Sigma, cat. no. A9941) was added. The reaction was terminated by adding the stop solution (3N HCl); the samples were subjected to absorbance reading at 450 nm wavelength (OD_450_) using the Synergy 2 multiplate reader (BioTek, Winooski, VT, USA). The normalized OD_450_ value of each sample in the 96-well plate was calculated by subtracting the OD_450_ value of the uncoated well from the OD_450_ value of the corresponding coated well. The cutoff OD_450_ value was set as the mean plus three standard deviations of the OD_450_ values obtained from the negative controls. The N-specific IgG endpoint titer was determined at the highest dilution to give an OD_450_ value exceeding the cutoff OD_450_ value, which was set as the mean plus four standard deviations of the OD_450_ value obtained from the negative samples that contain the secondary antibody alone.

### 4.7. Homemade N-Based Cat IgG ELISA

A similar ELISA method as described above was used to evaluate 31 cat serum samples that were determined to be either positive or negative for anti-N antibody seroprevalence through various assays [27]. Briefly, a 96-well MaxiSorp plate was either uncoated or coated with 100 ng of the purified N(+RNA) or N(−RNA) protein, in duplicate, incubated overnight at 4 °C, and blocked with 5% nonfat milk in PBST buffer for 1 h at 37 °C. Heat-inactivated cat sera were diluted to 1:50 with 1% nonfat milk in PBST, added equally to both uncoated and coated wells, and incubated for 1 h at 37 °C. Following washing with PBST, HRP-conjugated goat anti-cat IgG polyclonal antibody (Rockland Immunochemicals, Gilbertsville, PA, USA; cat. no. RL602-1302) was added at a 1:1000 dilution and incubated for 45 min at 37 °C. Following the addition of the substrate ABTS and stop solution (3N HCl), the absorbance at 450 nm wavelength (OD_450_) was measured using the Synergy 2 multiplate reader (BioTek). The normalized OD_450_ value of each sample was calculated by subtracting the OD_450_ value of the uncoated well from the OD_450_ value of the coated well. The cutoff OD_450_ value was set as the mean plus three standard deviations of the OD_450_ values obtained from the negative samples.

### 4.8. Cat Serum Samples

The seropositive and seronegative cat serum samples in this study were from our previously published study [27]. These pet cats were presented to the veterinary medical center for various medical conditions or wellness checks. At the time of admission or presentation, there was no known or perceived association of COVID-19 disease in these patients. As the animal sera used in this study were discarded and archived samples, we could neither determine the status of viral infection in animals at the time of admission by RT-PCR or IgM assay, nor were we aware of the owners’ health information. The serum samples were heat-inactivated at 56 °C for 30 min and stored at −20 °C until use.

## Data Availability

Data supporting reported results can be found at 10.6084/m9.figshare.15170625.
